# A hypothesis-driven approach identifies CDK4 and CDK6 inhibitors as candidate drugs for treatments of adrenocortical carcinomas

**DOI:** 10.18632/aging.101356

**Published:** 2017-12-26

**Authors:** Djihad Hadjadj, Su-Jung Kim, Thomas Denecker, Laura Ben Driss, Jean-Charles Cadoret, Chrystelle Maric, Giuseppe Baldacci, Fabien Fauchereau

**Affiliations:** ^1^ Pathologies de la Réplication de l'ADN, Université Paris-Diderot – Paris 7, Sorbonne Paris Cité, CNRS UMR7592, Institut Jacques-Monod, 75205 Paris Cedex 13, France; ^2^ ePôle de Génoinformatique, Université Paris-Diderot – Paris 7, Sorbonne Paris Cité, CNRS UMR7592, Institut Jacques-Monod, 75205 Paris Cedex 13, France

**Keywords:** cancer, CDK6, adrenocortical, palbociclib, ribociclib

## Abstract

High proliferation rate and high mutation density are both indicators of poor prognosis in adrenocortical carcinomas. We performed a hypothesis-driven association study between clinical features in adrenocortical carcinomas and the expression levels of 136 genes involved in DNA metabolism and G1/S phase transition. In 79 samples downloaded from The Cancer Genome Atlas portal, high *Cyclin Dependent Kinase 6* (*CDK6*) mRNA levels gave the most significant association with shorter time to relapse and poorer survival of patients. A hierarchical clustering approach assembled most tumors with high levels of *CDK6* mRNA into one group. These tumors tend to cumulate mutations activating the Wnt/β-catenin pathway and show reduced *MIR506* expression. Actually, the level of *MIR506* RNA is inversely correlated with the levels of both *CDK6* and *CTNNB1* (encoding β-catenin). Together these results indicate that high *CDK6* expression is found in aggressive tumors with activated Wnt/β-catenin pathway. Thus we tested the impact of Food and Drug Administration-approved CDK4 and CDK6 inhibitors, namely palbociclib and ribociclib, on SW-13 and NCI-H295R cells. While both drugs reduced viability and induced senescence in SW-13 cells, only palbociclib was effective on the retinoblastoma protein (pRB)-negative NCI-H295R cells, by inducing apoptosis. In NCI-H295R cells, palbociclib induced an increase of the active form of Glycogen Synthase Kinase 3β (GSK3β)responsible for the reduced amount of active β-catenin, and altered the amount of *AXIN2* mRNA. Taken together, these data underline the impact of CDK4 and CDK6 inhibitors in treating adrenocortical carcinomas.

## INTRODUCTION

Adrenocortical carcinomas (ACCs) are rare (annual incidence 0.5 to 2 patients per million individuals) but deadly cancers (the overall five-year survival of patients has been estimated below 35% in most studies) with limited opportunities of treatment [1,2]. In ACCs, indicators of high proliferation rate, such as an abnormal number of mitoses (>5 mitoses per 50 high power fields) and a high Ki-67 labeling index, constitute potent markers of poor prognosis [3–6]. This tendency has been confirmed by transcriptomic approaches that segregated ACCs into two groups. An overall overexpression of genes associated with cell proliferation has been observed in the group of most aggressive ACCs [7–9]. Abnormal expression of genes involved in DNA metabolism may also contribute to a higher mutation rate and thus to the acquisition of new cellular abilities, such as resistance to drugs, and the ability to relapse and to metastasize. In ACCs, mutation density has recently been associated with clinicopathological parameters such as overall survival time and time to recurrence [10].

Considering the central role of DNA metabolism in the evolution of cancers, we have tested the association of clinical parameters with the expression levels of 136 genes involved in the G1/S phase transition, DNA replication and response to DNA damage. This study was performed on transcriptomic data of 79 ACC patients shared by The Cancer Genome Atlas (TCGA) consortium [10]. The most significant association was obtained with the *Cyclin Dependent Kinase 6* (*CDK6*) gene, whose overexpression is associated with short time before tumor relapse and death of patients. We found that *CDK6* mRNA is overexpressed in a group of aggressive ACCs enriched in mutations in genes of the Wnt/β-catenin pathway.

Based on these results, we considered CDK6 inhibitors as potential candidates for therapy of ACCs. Palbociclib (PD-0332991, IBRANCE®, Pfizer), and ribociclib (LEE011, Kisqali®, Novartis) are both CDK4 and CDK6 (CDK4/6) inhibitors. Palbociclib is efficient in combination with letrozole (Femara®, Novartis) or fulvestrant (FASLODEX®, AstraZeneca) in patients with hormone receptor positive (HR+)-advanced breast cancers. It has recently been approved in the United States of America and the European Union in these combinations [11–14]. Ribociclib, in combination with letrozole, was recently approved by the Food and Drug Administration (FDA) as a frontline treatment for HR+ and human epidermal growth factor receptor 2 negative (HER2-)-advanced or metastatic breast cancers [15,16]. We thus characterized the impacts of these two FDA-approved CDK4/6 inhibitors on the cell cycle and survival of SW-13 and NCI-H295R cell lines as a first step to test their potential therapeutic properties against ACCs.

## RESULTS

### A hierarchical clustering of G1/S transition and DNA replication / repair genes identifies four transcriptional clusters

As a first step of our study on transcriptomic data related to the G1/S transition and DNA replication genes in ACCs, we established a list of 136 genes involved in these processes, based on ontology annotations in the Kyoto Encyclopedia of Genes and Genomes (KEGG) database [17] and bibliographic data (Supplementary Table 1). These genes could be classified into six groups based on their biological functions, namely G1/S transition, DNA polymerases, DNA replication, S phase checkpoint, stalled replication fork restart / double strand break repair, and dNTP synthesis. We added the expression levels of the *Marker of Proliferation Ki-67* (*MKI67*) gene, as its expression is a keystone marker of proliferation widely used in adrenal cancer prognosis. For these 137 genes (including *MKI67*), RNAseq data of ACCs from 79 patients were then downloaded from the TCGA portal.

To identify clusters of co-expressed genes, we first estimated the Pearson correlation coefficient of these 137 genes with each other, based on mRNA levels (Supplementary Figure 1). We then performed a hierarchical clustering of genes, in which the dissimilarity between gene clusters was calculated with the Pearson correlation values. Genes clustered into one group tended to have correlated mRNA levels. Hierarchical clustering produced four clusters of genes. Clusters 3 and 4 contain 56 (including *MKI67*) and 33 genes, respectively. The Pearson correlation test showed that the expression levels of each of the 55 genes of cluster 3 and 28 genes of cluster 4 (out of 33) are significantly correlated with the expression of *MKI67* and are associated with this classical marker of proliferation rate (Supplementary Figure 1 and Supplementary Table 1). These 83 genes are implicated in the six aforementioned functional processes. In particular, they include the genes encoding the replicative DNA polymerases ɑ, δ and ε, with the exception of the *POLD4* gene, which encodes the p12 accessory subunit of polymerase δ. Clusters 1 and 2 contain 23 and 25 genes, respectively. While the expression values in ACCs of 40 genes showed no significant correlation with *MKI67*, 9 genes in cluster 1 were inversely correlated with this proliferation marker. Among these is *POLD4*. The other inversely correlated genes include genes of negative cell cycle regulators (*CDKN1C*, *CCND2* and *RBL2*) and DNA repair genes (*ATM*, *RAD50*, *MCM9*, *RMI1* and *TOP3A*).

### *CDK6* expression shows significant prognostic value in ACCs

**Figure 1:**
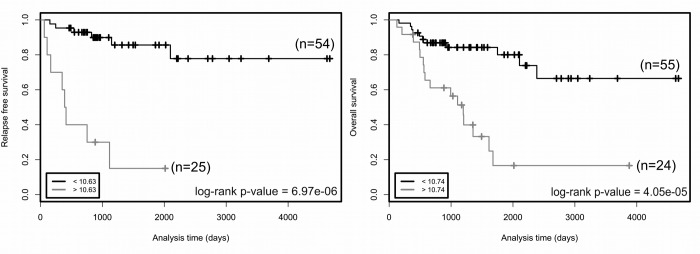
Relapse free survival (left panel) and overall survival (right panel) rates in groups of patients as a function of *CDK6* expression Two groups of patients with *CDK6* expression higher (grey curves) or lower (black curves) than cutoff values (bottom left in the graphs) have been defined for each trait. The number of patients in each group is indicated in parentheses. Cutoffs are expression values that maximized the significance of log-rank tests. *p* values of log-rank tests are at the bottom right in each box. Cutoff value is a gene-level transcription estimate, in RNA-Seq by Expectation Maximization (RSEM) normalized counts (downloaded from TCGA portal).

We then studied the association of the expression of the 137 genes with the overall survival (OS) and relapse free survival (RFS) of patients (Supplementary Table 1). Association was tested using the Log-rank test, which is routinely used to compare survival distributions of two groups of patients. Among the genes tested, the expression level of 114 genes was significantly correlated with OS, and that of 68 genes with RFS. Since proliferation is widely used in medical oncology, we focused our attention on the 28 genes associated with OS and/or RFS, but unrelated to *MKI67* (Table 1). Higher mRNA levels of genes encoding translesion DNA polymerases, namely *POLB, POLL, REV1* and *REV3L,* and lower expression of *POLK,* indicated poor prognosis (Table 1). Increased expression associated with poor prognosis was also observed for genes involved in E2F-dependent G1/S transition (*CDK6, CCND1, E2F3-5* and *TFDP2*), in DNA replication initiation (*ORC2L, ORC4L* and *ORC5L*), in S phase checkpoint (*TIPIN, TP53*) and stalled fork restart and double-strand break repair (*SMARCAL1* and *MUS81*). In contrast, associated with poor prognosis, we observed reduced gene expression of inhibitors of the E2F pathway (*CDKN2B, HDAC1, RB1*), of genes involved in DNA replication (*GINS3* and *TOP1*), in S phase checkpoint (*RAD17, NBN* and *TP53BP1*), and in dNTP synthesis (*RRM2B*). The gene with the most significant Log-rank test for RFS is *CDK6* (cutoff value > 10.63, n=25 out of 79 patients, adjusted *p* value = 6,97 × 10^−6^). Its expression is also significantly associated with OS (cutoff value > 10.74, n=24 out of 79 patients, adjusted *p* value = 4.05 × 10^−5^). *CDK6* and 9 other genes unrelated to proliferation, namely *E2F3, E2F5, ORC2L, ORC4L, ORC5L, CDKN2B, POLG2, REV3L* and *SMARCAL1*, belong to the expression cluster 2 (Supplementary Figure 1) and thus have similar expression profiles in ACC patients. The Kaplan-Meier analyses demonstrate a shorter time of OS and RFS of patients associated with high *CDK6* expression (Figure 1). We confirmed the association between the *CDK6* transcription level and shorter time to relapse and death using the Log-rank test on previously published data from a French cohort [18]. In this sample, patients with levels higher than the cutoff values again showed shorter times to relapse (*p* value = 0.041, cutoff value > 5.067, n=38 out of 44 patients) and death (*p* value = 1.51 × 10^−6^, cutoff value > 6.027, n=19 out of 44 patients).

**Table 1:** Association between the expression levels of 28 genes with time of RFS and/or OS, but not with the expression of the *MKI67* gene in ACC tumor samples

		Correlation with MKI-67		Relapse Free survival					Overall Survival				
Gene	Cellular process	coef. Correl.	P-value	Risk group	Cutoff	Percentile	P-value	Adj. P-val.	Risk group	Cutoff	Percentile	P-value	Adj. P-val.
**DNA polymerases**													
POLB	Replication / repair	0.0238	0.8268	-	7.692	9.3	0.1461	0.1629	High	8.705	77.2	0.0010	**0.0018**
POLG2	Mitochondrial DNA replication	0.1385	0.201	High	7.394	88.9	0.0058	**0.0234**	Low	7.27	87.3	0.0240	**0.0303**
POLL	Replication / repair	−0.06111	0.5738	High	9.842	81.5	0.0128	**0.0352**	-	9.327	45.6	0.1071	0.1165
REV3L	Translesion DNA synthesis	0.149	0.1685	-	8.224	29.6	0.1252	0.1408	High	9.295	86.1	0.0024	**0.0039**
REV1	Translesion DNA synthesis	0.2021	0.06062	High	9.479	44.4	0.0122	**0.0352**	High	10.22	77.2	7.64E-06	**1.89E-05**
POLK	Translesion DNA synthesis	−0.1658	0.125	Low	9.145	29.6	0.0008	**0.0075**	Low	8.899	26.6	1.26E-05	**2.90E-05**
**G1/S checkpoint**													
CDK6	CDK and their regulators	0.1239	0.2529	High	10.63	79.6	5.12E-08	**6.97E-06**	High	10.74	69.6	1.79E-05	**4.05E-05**
CDKN2B	CDK and their regulators	0.1209	0.2645	Low	7.09	29.6	0.0031	**0.0169**	High	8.264	75.9	0.0025	**0.0039**
CCND1	CDK and their regulators	0.02788	0.7976	-	12.53	87.0	0.0266	0.0510	High	12.66	81.0	0.0038	**0.0056**
HDAC1	pRB pathway	0.1997	0.06391	Low	11.03	48.1	0.0164	**0.0399**	Low	10.31	12.7	0.0102	**0.0140**
RB1	pRB pathway	−0.1955	0.06975	Low	9.334	13.0	0.0076	**0.0285**	-	9.899	29.1	0.0556	0.0652
E2F3	pRB pathway	0.1764	0.1024	High	9.201	87.0	0.0130	**0.0352**	-	7.804	11.4	0.1264	0.1364
E2F4	pRB pathway	0.1165	0.2827	Low	10.96	61.1	0.0217	**0.0440**	-	10.77	40.5	0.1294	0.1375
E2F5	pRB pathway	0.08457	0.436	High	5.532	20.4	0.0411	0.0636	High	7.213	84.8	0.0107	**0.0146**
TFDP2	pRB pathway	−0.007805	0.9428	High	8.354	72.2	0.0049	**0.0209**	-	7.35	13.9	0.1284	0.1375
**DNA replication**													
ORC2L	Pre-replication complex	0.137	0.2056	High	8.245	77.8	0.0040	**0.0190**	High	8.577	84.8	2.69E-05	**5.91E-05**
ORC4L	Pre-replication complex	−0.05241	0.6296	High	8.576	29.6	0.0104	**0.0352**	-	9.099	75.9	0.0518	0.0612
ORC5L	Pre-replication complex	0.1365	0.2076	-	8.235	16.7	0.2891	0.2912	High	9.51	88.6	0.0043	**0.0063**
GINS3	Initiation of DNA replication	0.01906	0.8609	-	9.355	57.4	0.0819	0.1032	Low	8.902	41.8	0.0024	**0.0038**
TOP1	Topoisomerase	−0.03081	0.7769	Low	10.86	25.9	0.0090	**0.0314**	Low	10.55	19.0	0.0146	**0.0195**
**S phase checkpoint**													
RAD17	ATR + Rad17-9-1-1 DNA damage sensors	−0.132	0.2229	-	9.195	25.9	0.0477	0.0684	Low	9.471	49.4	0.0005	**0.0008**
NBN	ATM - MRN DNA damage sensors	−0.04222	0.6977	Low	9.129	9.3	0.0034	**0.0179**	Low	10.39	79.7	0.0175	**0.0225**
TIPIN	ATM/ATR pathways mediators	0.1816	0.09256	-	5.981	40.7	0.2053	0.2181	High	6.53	70.9	0.0054	**0.0077**
TP53	ATM and ATR pathways effector	−0.06086	0.5754	High	10.02	79.6	0.0002	**0.0040**	High	10.13	84.8	0.0019	**0.0032**
TP53BP1	ATM and ATR pathways effector	−0.09276	0.3927	-	9.333	11.1	0.1691	0.1855	Low	10.16	57.0	0.0168	**0.0217**
**Stalled forks restart by remodeling / DSB repair**													
SMARCAL1	Helicase	0.146	0.1773	High	8.843	35.2	0.0205	**0.0428**	High	9.37	81.0	0.0152	**0.0200**
MUS81	Holliday junction resolution	0.1526	0.1582	High	8.886	66.7	0.0834	0.1040	High	8.951	59.5	4.94E-06	**1.27E-05**
**dNTP synthesis**												
RRM2B	Ribonucleotide reductase	−0.1183	0.2751		10.89	70.4	0.0296	0.0523	Low	9.512	17.8	0.0004	**0.0007**

RNAseq and clinical data of n=54 and n=79 ACC samples were downloaded from the TCGA website and used for the Log-rank correlation tests for RFS and OS, respectively. The Log-rank test was used to compare survival distribution of two groups of patients, considering that their gene expression values were higher or lower than a cutoff. For each gene, a succession of Log-rank tests was performed with all possible cutoff values, given gene expression levels in tumors. The cutoff value chosen to segregate the “high” and “low” expression groups of patients was the one that maximized the significance of Log-rank tests. The percentile is the proportion of individuals below the cutoff value. “Adj. P-val.” are the *p* values of the Log-rank tests that have been adjusted following the Benjamini Hochberg method (in bold when significant). Coeff. correl. is the Pearson product-moment correlation coefficient estimated between the expression level of each gene with *MKI67*. The risk group is given when the association with RFS and/or OS was significant. High and Low risk groups indicate the group with the worst prognosis, based on their expression level, higher or lower than the cutoff, respectively.

### Molecular and clinical features of patients with high expression of *CDK6*

**Figure 2:**
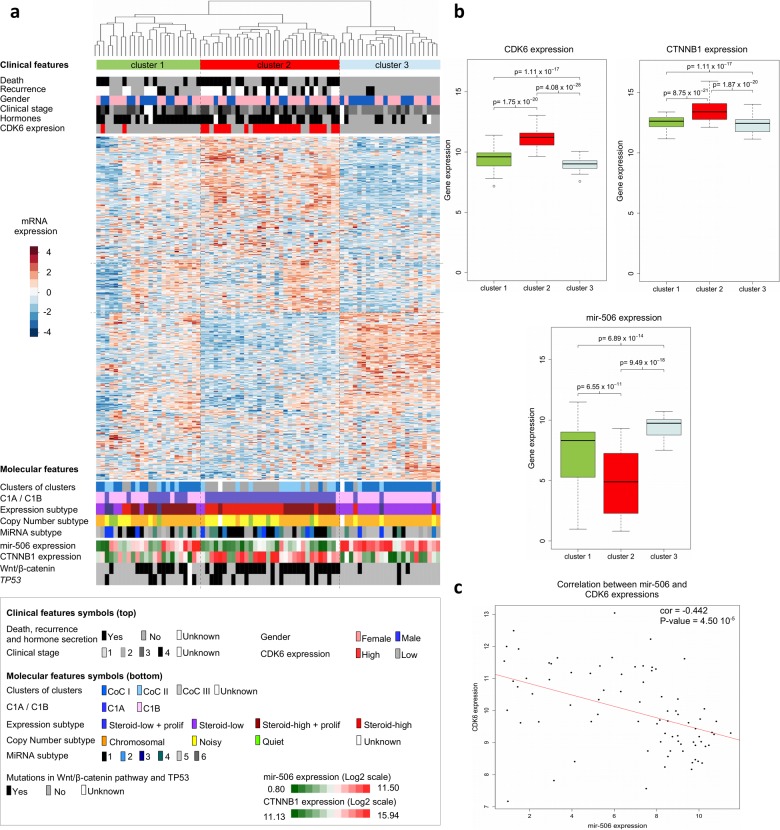
Clinical and molecular features of adrenocortical carcinomas (**a**) Hierarchical clustering of the 79 ACC samples results into clusters 1, 2 and 3, as indicated in color bars at the top of the heatmap. Clustering is based on the expression levels of the top 500 most variant genes from the transcriptomes of the 79 ACCs. Dissimilarities between samples are indicated by the dendrogram at the top of the heatmap. Expression levels are shown by colors. Colors follow the base-2 log color scale at the left of the heatmap. The color symbols for clinical and molecular features are indicated in the frame at the bottom of the heatmap. (**b**) Box-plots showing the distribution of the *CDK6*, *CTNNB1* and *MIR506* gene expression values of tumor samples in the three transcriptome-based clusters. A base-2 log scale is used for the Y-axis showing gene expression. The band at the middle of the box indicates the median value. The bottom and top of the box are the 25^th^ and 75^th^ percentiles. Bottom and top whiskers represent the limits of exclusion of outliers. *p* values show the significance of the unilateral Wilcoxon-Mann-Whitney test. (**c**) Scatter plot showing the expression values of *CDK6* and *MIR506* of ACC samples, for which a base-2 log scale is used. The value of the Pearson coefficient (cor.) and the Pearson test *p* value for correlation are indicated at the top-right of the scatter plot. The red line is the linear regression line illustrating the negative correlation of expression between *CDK6* and *MIR506*. Clinical and molecular features (with the exception of the CDK6 expression group) were previously described [9,11].

Since our cell cycle / DNA metabolism approach highlighted the association of high *CDK6* expression with short times to relapse and death, we looked for other clinical and molecular features shared by patients showing *CDK6* overexpression. Hierarchical clustering based on mRNA levels of the 500 most variant genes in ACCs led to the constitution of clusters designated 1, 2 and 3. These clusters as a whole reflect the mRNA-based classification (Clusters of Clusters) recently published by TCGA [10] (Figure 2a). Cluster 2 includes 23 out of the 25 “*CDK6-*high” samples. The average expression level of *CDK6* is higher in cluster 2 than in cluster 1 or 3 (cluster 2 vs 1, *p* value = 1.75 × 10^−20^, cluster 2 vs 3, *p* value =4.08 × 10^−28^) (Figure 2b). Cluster 2 tumors contain the majority of Cluster 1A (C1A) previously classified samples with a high production of steroids. A clinical feature significantly associated with the *CDK6* mRNA level is the synthesis of hormones, that are known to be an indication of poor prognosis in ACC patients [19] (Table 2). Cluster 2 also includes the majority of mutations and copy number variations that activate the Wnt/β-catenin signaling pathway. The microRNA-based clustering recently published by TCGA has led to a new classification in six groups [10]. Since the data involved *microRNA506* (*MIR506*) in the regulation of both *CDK6* and *CTNNB1* (encoding β-catenin), we analyzed its expression level in the 79 ACC samples. The expression of microRNA 506 was significantly lower in cluster 2 compared to clusters 1 and 3 (cluster 2 vs 1, *p* value = 6.55 × 10^−11^, cluster 2 vs 3, *p* value = 9.49 × 10^−18^) (Figure 2b). Correlation analyses revealed an inversed ratio between the *CDK6* and *MIR506* expression levels (Figure 2c, test for correlation based on Pearson's product moment coefficient, coefficient = −0.442, *p* value = 4.50 × 10^−5^). The anti-correlation between *MIR506* and *CTNNB1* RNAs has been previously described by TCGA [10]. Thus, a low *MIR506* expression level could contribute to higher levels of both *CDK6* and *CTNNB1* mRNAs in ACCs.

**Table 2:** Correlation of clinical features with the *CDK6* gene expression level of ACC tumors of n=79 TCGA patients

	High CDK6	Low CDK6	*p* value
**Age**	47.6 [40.2;55.0]	46.2 [42.2;50.2]	0.747
**Tumor Size**	139 [108;169]	125 [96;154]	0.531
**Gender**			0.132
Male	6	25	
Female	18	30	
**Hormonal Secretion**			**0.038**
Yes	19	29	
No	4	22	
**Laterality**			0.468
Right	9	26	
Left	15	29	
**Clinical stage**			0.056
I	0	9	
II	10	26	
III	7	9	
IV	7	8	
**Weiss score**			0.491
<4	4	10	
4-5	7	9	
6-7	3	13	
>7	5	9	
**Recurrence**			6.97 × 10^−6^
Yes	7	7	
No	2	37	
Time to recurrence	689 [ 297;1080]	1435 [1117;1753]	
**Death**			4.05 × 10^−5^
Yes	16	10	
No	8	42	
Time to death	1059 [746;1372]	1495 [1210;1779]	

The “High” and “Low” CDK6 expression groups are based on the results of the Log-rank test for relapse-free survival. Fisher's exact test was used to test the independence of discrete clinical features (gender, hormonal secretion, laterality and clinical stage) from expression level. Independence was rejected only for hormonal secretion, with an enrichment of hormone secretion among the “High” CDK6 expression group (true odds ratio = 3.5454, 95% confidence interval [0.981;16.385]). *p* values given for Recurrence and Death traits are those of the Log-rank test. Time to recurrence and death are the average values estimated from “High” CDK6 and “Low” CDK6 groups.

### Palbociclib and ribociclib lower cell viability of the SW-13 and NCI-H295R cell lines

Palbociclib and ribociclib inhibit CDK4/6 and are used for the treatment of breast cancer [11,12,15,16]. We tested the effects on cell viability of CDK4/6 inhibitors either with or without mitotane, a well-known adrenolytic drug that is currently used to treat ACCs. Viability was measured using SW-13 and NCI-H295R cells.

**Figure 3:**
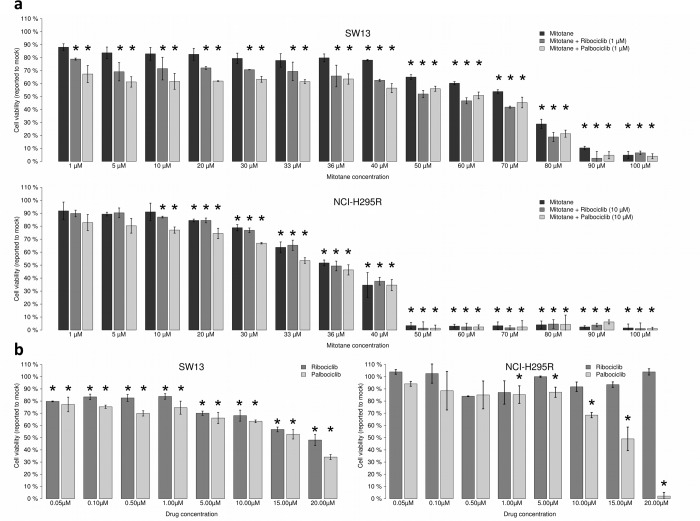
Ribociclib and palbociclib lower viability of SW‐13 and NCIH295R adrenocortical cell lines Cells were treated with drugs during 96 h. (**a**) Mitotane alone (from 1 μM to 100 μM) or combined with CDK inhibitors dose‐response bar graphs on SW‐13 and NCIH295R cells. CI50 = 50% Combination indexes. (**b**) Ribociclib and palbociclib dose‐response bar graphs on SW‐13 (left panel) and NCIH295R cells (right panel). Drug concentration ranges from 0.05 μM to 20 μM. For each measurement error bars indicate standard deviation value, estimated from two different experiments. Asterisks show the significant t‐tests (*p* values < 0.05).

Mitotane was first tested alone. It decreased SW-13 and NCI-H295R viability with an IC50 (concentration needed to reduce viability to 50%) of 68.38 μM and 33.16 μM, respectively (Figure 3a). As previously shown, SW-13 cells are less sensitive to mitotane than NCI-H295R cells [20]. We then combined increasing concentrations of mitotane with either 1 μM of palbociclib or 1 μM of ribociclib on SW-13 and 10 μM of palbociclib or ribociclib on NCI-H295R cells (concentration of ribociclib or palbociclib inducing a 20% reduction of viability when they are used alone). In SW-13 cells, both drugs showed an additive effect with mitotane, with a 50% combination index of 0.997 for mitotane with palbociclib and of 1.043 for mitotane with ribociclib (Figure 3a). However, in NCI-H295R cells, mitotane showed an additive effect only with palbociclib with a combination index of 1.021 (Figure 3a). Mitotane combined with ribociclib showed no difference on cell viability compared to the effect of mitotane alone (Figure 3a).

The effects of palbociclib and ribociclib alone on both the SW-13 and NCI-H295R cell lines were also tested. Both drugs decreased cell viability in the SW-13 cell line, with an IC50 = 15.50 μM for palbociclib and an IC50 = 19.08 μM for ribociclib (Figure 3b). In NCI-H295R cells only palbociclib had an effect on cell viability with an IC50 = 14.06 μM (Figure 3b). After treatment with 20 μM palbociclib cell viability was estimated to be close to 0%. Hence, palbociclib is the only drug active on both cancer cell lines and it strongly affects cell viability of NCI-H295R cells.

### Palbociclib induces cell cycle arrest and senescence in SW-13 and NCI-H295R cell lines

**Figure 4:**
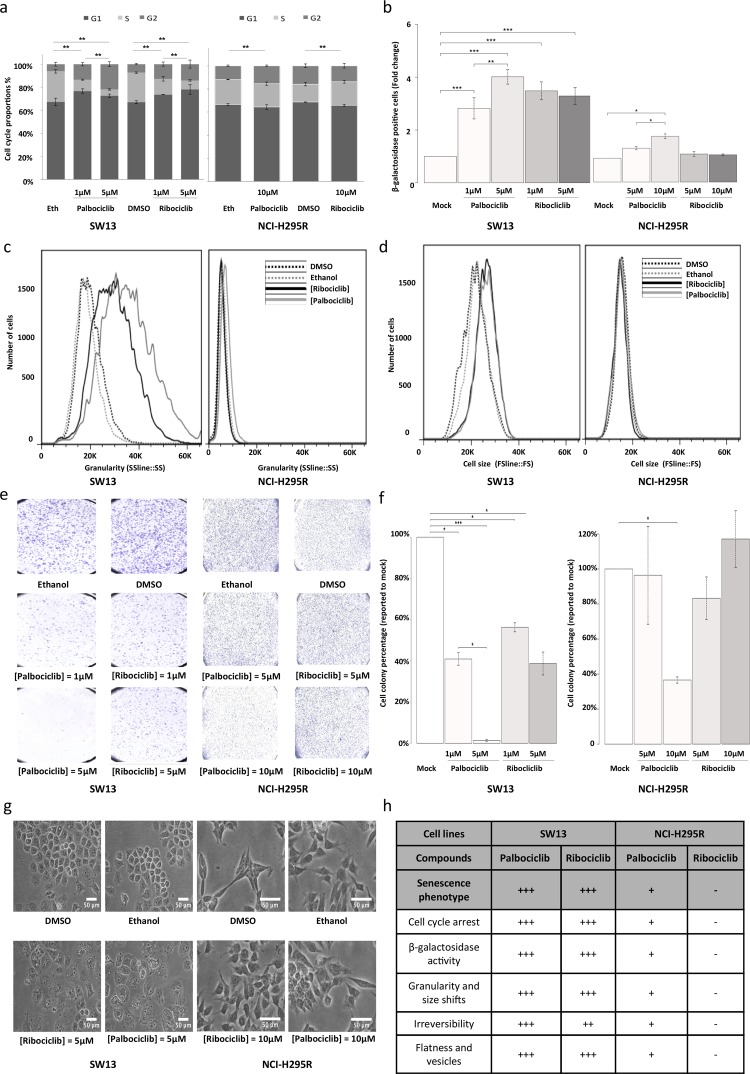
Senescence features induced by ribociclib and palbociclib (**a**) Cumulative bar chart showing the proportion of SW-13 and NCI-H295R treated cells in G1, S and G2/M cell cycle phases. (**b**) β-galactosidase activity is used as marker of senescence. The number of cells with β-galactosidase activity after treatment with ribociclib or palbociclib was counted and related to the number of cells with β-galactosidase activity after treatment with the vehicle only (DMSO or ethanol, respectively). Mean ratio and standard deviation were estimated from three independent experiments. (**c**) Flow cytometry analyses showing granularity of SW-13 and NCI-H295R cells treated with either palbociclib or ribociclib. Granularity is estimated by measuring the Side Scatter values (on the X-axis). (**d**) Flow cytometry analyses showing cell size of SW-13 and NCI-H295R treated with either palbociclib or ribociclib. Cell size is estimated by measuring the Side Scatter values (on the X-axis). (**e**) Colonies formed by SW-13 and NCI-H295R cells after coloring with crystal violet during the clonogenic assay. (**f**) The number of cell colonies formed after treatment with ribociclib or palbociclib was counted and related to the number of colonies formed after treatment with the vehicle only (DMSO or ethanol, respectively). The mean and standard deviation of percentage of colonies (compared to mock treatment) were estimated with three independent experiments. (**g**) Images in phase contrast showing the change of cell morphology of SW-13 and NCI-H295R cells upon treatment with either palbociclib or ribociclib. (**h**) Table summarizing the main aspects of senescence in both cell lines when treated with either palbociclib or ribociclib. For (**b**) and (**f**), ^*^P<0.05, ^**^P<0.01, ^***^P<0.001.

To better characterize the effects of the two CDK4/6 inhibitors on the viability of the SW-13 and NCI-H295R cells, the cell cycle of both cell lines upon treatment with either palbociclib or ribociclib was investigated. In SW-13 cells, 1 and 5 μM of both inhibitors induced cell cycle arrest, with a smaller proportion of cells in the S phase and an increased proportion of cells in the G1 and G2 phases, when compared with mock-treated cells (Figure 4a and Supplementary Figure 2a). The cycle of NCI-H295R cells was also affected by drug treatments (Figure 4a and Supplementary Figure 2a). 10 μM Palbociclib increased the proportion of cells in G2 phase, whereas 10 μM ribociclib increased the proportion of cells in S phase.

We tested whether reduced cell viability was associated with senescence. In SW-13 cells treated with palbociclib or ribociclib, we observed a significant increase in the percentage of cells harboring β-galacto-sidase activity, an indicator of senescence (Figure 4b and Supplementary Figure 2b). Both treatments also induced higher cell granularity and increased cell size in flow cytometry analyses (Figures 4c and d).

Induction of vesicle formation and increased flatness were also observed with bright-field microscopy (Figure 4g). Reversibility of the cell cycle arrest was tested by clonogenic assay. Ribociclib (1 and 5 μM, *p* values = 0.001 and 0.011, respectively), and palbociclib (1 and 5 μM, *p* values = 0.022 and 0.6 × 10^−3^, respectively) significantly decreased the ability of SW-13 to form clones, with clonogenic ability close to 0 after treatment with 5 μM palbociclib (Figures 4e and f). Taken together, these observations indicate induction of senescence in SW-13 cells after treatment with either palbociclib or ribociclib. In NCI-H295R cells, only palbociclib induced a significant increase of β-galacto-sidase activity (Figure 4b) when compared to mock-treated cells. In contrast with our observations using SW-13 cells, no marked increase of cell size was detected in NCI-H295R cells treated with either palbociclib or ribociclib (Figure 4d). Only a slight shift of cellular granularity was observed when NCI-H295R cells were treated with palbociclib, but not with ribociclib (Figure 4c). Finally, only 10 μM palbociclib induced a significant irreversibility of cell cycle arrest, as tested by clonogenic assay (*p* value = 0.013). Thus, NCI-H295R cells treated with palbociclib show some features of senescence, but less pronounced than SW-13 cells (Figure 4h).

**Figure 5:**
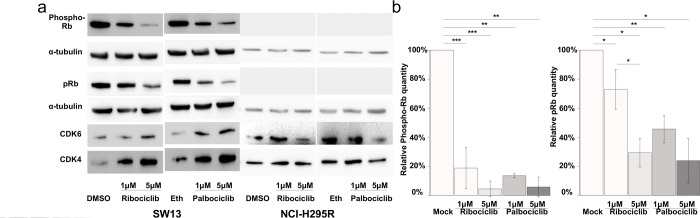
CDK4/6 inhibitors lower the proportion of Phosphorylated-pRB and the total amount of pRB proteins in SW-13 cells (**a** and **b**) total pRB protein, phosphorylated-RB (phospho-RB), CDK6 and CDK4 were detected by western blot. (**a**) The results present the relative amounts of either Phospho-RB or pRB in drug-treated cells, compared to Phospho-RB or pRB in mock-treated cells. Values are the mean and standard deviations of two independent experiments. For the western blot experiments, ɑ-tubulin was used as a loading control. In (**b**), significance was tested with the t-test. ^*^P<0.05, ^**^P<0.01, ^***^P<0.001.

Since ribociclib and palbociclib inhibit CDK4/6, they could impair the phosphorylation of the Retinoblastoma protein pRB, a crucial step in the G1/S transition. Consequently, we evaluated the levels of CDK4, CDK6, pRB and phosphorylated-pRb (Phospho-Rb) in such drug-treated cells (Figure 5a). In SW-13 cells, the amount of both CDK4 and CDK6 proteins increased after treatment with palbociclib or ribociclib. Such treatments also significantly lowered the amounts of both Phospho-Rb and pRB (Figures 5a and b). These experiments thus show that CDK4/6 inhibition following palbociclib or ribociclib treatment reduces the amount of Phospho-RB in SW-13 cells, and is associated with a senescence-like phenotype. pRB was not detected in NCI-H295R protein extracts (Figure 5a), which is consistent with the fact that this cell line carries a homozygous deletion of the *RB transcriptional corepressor 1* (*RB1*) gene (COSMIC mutation ID: 19554, c.862_2787del1926) [21]. This deletion could possibly hamper the action of CDK4/6 inhibitors on this cell line, in which only a slight senescence-like phenotype was observed.

### Palbociclib targets the Wnt/β-catenin pathway and induces apoptosis in NCI-H295R cells

**Figure 6:**
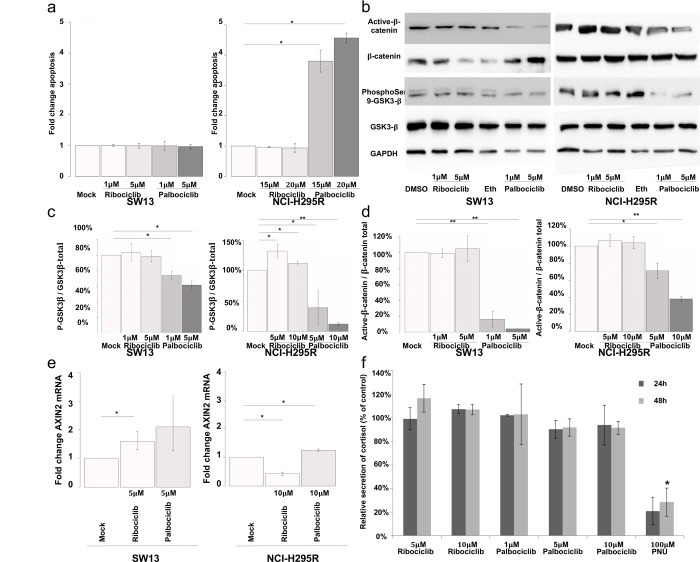
Palbociclib induces apoptosis in NCI-H295R cells by targeting the Wnt/β-catenin pathway (**a**) Bar graphs showing apoptosis fold change in SW-13 or NCI-H295R cells upon treatment with either palbociclib or ribociclib. Caspase 3/7 activity was used as a read out to measure apoptosis. Results are the mean and standard deviations estimated based on three independent experiments. (**b**) Active β-catenin, total β-catenin, phospho-Ser9-GSK3β and total GSK3β were detected by western blot upon treatment with either palbociclib or ribociclib in both cell lines. GAPDH was used as a loading control. (**c** and **d**) Bar graphs showing the relative amount of phospho-Ser9-GSK3β on total GSK3β (c), or active β-catenin over total β-catenin (**d**) in both cell lines. Results are the mean and standard deviations based on two independent experiments. (**e**) Bar graphs showing levels of *AXIN2* mRNA upon treatment with either palbociclib or ribociclib, in both cell lines. The values indicate the fold-change of mRNA levels after treatment with palbociclib or ribociclib, compared to mock-treated cells. *AXIN2* mRNA levels in each condition were normalized to *ACTNB* (β-Actin) mRNA levels. qPCR were performed in triplicate. Mean and standard deviation are based on three different experiments. (**f)** Bar graph showing levels of secreted cortisol in the culture medium of NCI-H295R cells treated with ribociclib or palbociclib for 24 h and 48 h. Values are reported as percentage of concentration assayed with mock-treated cultures. Assay was performed in duplicate on culture media extracted from three different experiments. In (**a**), (**c**) and (**d**) Values are the mean ratio estimated from independent experiments. In (**a**), (**c**) and (**d**) significance was tested with t-test. In (**e**) and (**f**), significance was tested with the paired t-test. ^*^P<0.05, ^**^P<0.01.

Since palbociclib induced a significant decrease of viability of pRB negative NCI-H295R cells (Figure 3b), we evaluated its pro-apoptotic activity. SW-13 cells treated with either palbociclib or ribociclib showed no detectable apoptotic activity (Figure 6a). In NCI-H295R cells, an increase of apoptosis was detected after treatment with 20 μM palbociclib, but not with ribociclib (Figure 6a). This effect, specific of palbociclib, might be explained by the larger spectrum of kinases that it targets, compared with ribociclib. Additional palbociclib targets include GSK3βand its regulator AKT serine/threonine kinase [22]. Thus, the impact of palbociclib and ribociclib on the phosphorylation of GSK3β, in the SW-13 and NCI-H295R cell lines was also tested (Figures 6b and c). In SW-13 cells, only palbociclib reduced the ratio of Serine9-phosphorylated-GSK3β (inactive form) to total GSK3β (inactive pGSK3β / total pGSK3β)(Figures 6b and c). In NCI-H295R cells, 5 and 10 μM palbociclib significantly reduced the inactive pGSK3β / total pGSK3β ratio (Figures 6b and c). Ribociclib showed effects opposite to palbociclib, as it increased this ratio. GSK3β was previously shown to phosphorylate β-catenin and consecutively induce its degradation by proteasome. Since loss of β-catenin-dependent transcription was associated with apoptosis in NCI-H295R cells [23], the effects of palbociclib and ribociclib on the amount of β-catenin and on the level of *AXIN2* transcripts (a β-catenin-dependent transcription target) were examined (Figures 6b, 6d and 6e). Ribociclib did not significantly modify the amount of β-catenin in SW-13 and NCI-H295R cells. Nevertheless, the expression of *AXIN2* was significantly altered in both cell lines, through β-catenin-independent regulations. On the con-trary, palbociclib significantly lowered the amount of β-catenin in both cell lines. Palbociclib treatment also increased the level of *AXIN2* transcripts, as expected after inhibition of β-catenin-dependent transcription [23].

A previous analysis showed that apoptosis induced by PNU-74654 (an inhibitor of the T cell factor (Tcf)/β-catenin complex) was preceded by reduction of steroid secretion. We assayed cortisol concentration in the supernatant of NCI-H295R cells (Figure 6f) treated with either ribociclib (5 or 10 μM) or palbociclib (1, 5 or 10 μM). Assays were performed at the times of treatments that precede apoptosis (24 h and 48 h). PNU-74654 (100 μM) was used as a positive control, as this inhibitor of Tcf/β-catenin interaction effectively reduced secretion of cortisol and other steroids by NCI-H295R cells [23]. Actually, neither ribociclib nor palbociclib decreased cortisol secretion at concentrations and time-points tested (Figure 6f). Yet, 100 μM PNU-74654 decreased cortisol production by 80% after 24 h treatment (*p* value = 0.14) and by 72% after 48 h treatment (*p* value = 6.7 × 10^−4^). These effects of PNU-74654 (Figure 6f) are comparable to those previously observed [23]. Thus, Palbociclib-induced apoptosis of NCI-H295R cells is not preceded by a reduction of cortisol secretion.

Together, these results show that palbociclib-induced apoptosis is associated with a remarkable reduction of the amount of β-catenin and alters β-catenin-dependent transcription. Treatment with palbociclib could have potential benefits for the treatment of ACCs with an activated Wnt/β-catenin pathway.

## DISCUSSION

In this study we first classified 136 genes (Supplementary Table 1) involved in DNA replication/repair into four groups, according to their mRNA levels. A set of 83 genes overlapping clusters 3 and 4 showed a significant correlation with *MKI67*, a marker commonly used for proliferation in histology-based diagnosis of ACCs. Cluster 3 also includes the *POLQ* gene, encoding the translesional DNA polymerase Polθ (involved in DNA repair and in DNA replication timing program) [24,25], together with homologous recombination repair (HR) genes (namely *BRCA1*, *FANCD2*, *BLM* and *RAD51*). Positive correlations between expression levels of *POLQ* and HR genes have recently been reported in lung, breast and colorectal cancers [26]. The authors suggested that an expression reprogramming involving these genes could prevent genetic instability in a cancer context. Indeed, we observed a similar correlation between *POLQ* and HR genes in ACCs. These genes are also associated with the *MKI67* proliferation marker (Supplementary Figure 1). In ACCs, overexpression of *POLQ* and HR genes could contribute to genomic stability in highly proliferating tumors, possibly through DNA repair processes. mRNA levels of 28 genes in clusters 1 and 2 are associated with shorter time of relapse-free survival and overall survival, and are also independent of the cell proliferation marker *MKI67* cell proliferation. Thus, they may provide additional information in the molecular characterization of ACCs (Table 1). A positive correlation with shorter time to relapse of high mRNA levels of *POLB, POLL, REV1* and *REV3L*, and of low mRNA levels of *POLK* was noted. These genes encode translesion DNA polymerases, which can perform DNA synthesis despite the presence of DNA lesions. Altered gene expression and mutations affecting translesion polymerases have been observed in a variety of tumors and have been suggested to act as biomarkers in response to treatments [27–31]. The ability of translesion polymerases to perform synthesis despite DNA lesions contributes to resistance to DNA damaging treatments, and previous analyses have shown that their inhibition sensitizes tumors to chemotherapeutic agents [31–36]. Translesion polymerases are also error-prone, and thus can contribute to mutagenesis in tumors and progression of cancers [37]. Our analyses show that abnormal gene expression of translesion DNA polymerases is indeed a marker of poor prognosis independent of proliferation in ACCs. The development of small molecules targeting translesion DNA synthesis could potentially be beneficial for ACC patients with tumors expressing high levels of mRNAs encoding POLβ, POLλ, REV1 and REV3L translesion DNA polymerases.

In the second part of this study, we focused our analyses on the *CDK6* gene. CDK6 shares with CDK4 the ability to phosphorylate pRB and to induce the transition to S-phase of the cell cycle through the E2F-dependent transcription program. These cell functions are shared by CDK4 and CDK6 but we observed no correlation between the mRNA levels encoding these two kinases. Indeed, a significant correlation with overall survival and time to relapse has only been observed for the *CDK6* mRNA level (Table 1).

Since high expression levels of *CDK6* are associated with poor prognosis in ACCs (Figures 1 and 2), the impact of CDK6 inhibitors on the SW-13 and NCI-H295R cell lines was evaluated (Figure 3). While NCI-H295R cells are classically used as *ex vivo* models of adrenocortical carcinoma, the origin of SW-13 is contested. SW-13 cells were derived from a small cell carcinoma in the adrenal cortex [38]. However, these cells secrete no steroids and it is unclear whether they were derived from a primary tumor in the adrenal cortex or from a metastasis [39]. Keeping in mind the discussions concerning the origin of SW-13 cells, we chose to study the mechanisms reducing cell viability in this cell line in parallel with the pRB negative-NCI-H295R cells. SW-13 cells are highly sensitive to both palbociclib and ribociclib (Figure 3). Both drugs reduced SW-13 cell viability through an irreversible cell cycle arrest with a reduced proportion of cells in S-phase (Figure 4a). We also observed senescence features similar to those previously described in SW-13 cells [40]: β-galactosidase activity, enlarged and flattened cells and high granularity (Figures 4b, 4c, 4d and 4h). Ribociclib being a highly specific inhibitor of CDK4/6, the senescence phenotype is probably induced by deregulation of their effector proteins, such as pRB that plays a pivotal role, as it regulates both G1/S transition and induction of senescence. Such a hypothesis is consistent with the reduction of Phospho-Rb observed in treated cells (Figure 5). However, the senescence phenotype was more pronounced in palbociclib-treated cells and we could not exclude the involvement of other targets. In contrast with SW-13 cells, NCI-H295R cells showed resistance to ribociclib, and a higher IC50 value for palbociclib when compared to SW-13 cells (Figure 3b). A homozygous deletion in the *RB1* gene was previously described [21], and we confirmed the absence of the pRB protein in NCI-H295R extracts (Figure 5). The absence of pRB is probably involved in the NCI-H295R resistance to ribociclib, and also the relative resistance to palbociclib, as was observed in different types of cancers [41–44]. This resistance to CDK4/6 inhibitors caused by pRB loss of function would concern 6.8% and 7% of ACC patients, as estimated with the TCGA and the French cohorts of patients respectively [10,18]. Thus, a majority of ACC patients might benefit from CDK4/6 inhibitors.

While only a slight increase of senescence features was observed at low concentrations of palbociclib (Figure 4b), apoptosis explained the reduction in viability in NCI-H295R cell line detected at >10 μM (Figure 6a). In our effort to determine the cellular mechanism causing apoptosis in these pRB negative cells, we tested the impact of treatments on the activity of GSK3β. This kinase and its regulator AKT are targets of palbociclib and GSK3β phosphorylates β-catenin, leading to β-catenin degradation by the proteasome. Indeed, a higher ratio of active GSK3β and a reduction of the β-catenin active form were observed after treatment with palbociclib (Figures 6b, c and d). Furthermore, the transcriptional activity of β-catenin, was estimated with the level of *AXIN2* mRNA. Actually, increased transcription activity was observed after treatment with palbociclib (Figure 6e). Our results are consistent with the higher *AXIN2* mRNA level observed in NCI-H295R cells after treatment with PNU-74654, an inhibitor of Wnt/β-catenin signaling [23]. Moreover, this Wnt/β-catenin signaling inhibitor increased apoptosis in NCI-H295R cell cultures. Taken together, our observations suggest that palbociclib induces a strong reduction of active β-catenin, leading to aberrant transcription of β-catenin targets and to apoptosis. Besides its impact on β-catenin-dependent transcription, PNU-74654 also decreased steroid hormone secretion by NCI-H295R cells, as early as after 24 h of treatment, at con-centrations higher than 50 μM [23]. This reduction preceded the decreased of viability (only after 72 h treatment with 50 μM PNU-74654) and was supposed to result partially from the reduction in gene expression of *SF1* and *CYP21A2* genes. While we confirmed that 100 μM PNU-74654 decreased cortisol secretion, we observed no effect of palbociclib and ribociclib on the concentration of cortisol in the medium, after 24 h and 48 h of treatments (Figure 6f). Actually, PNU-74654 results in direct inhibition of β-catenin-dependent transcription that might cause the reduction of cortisol secretion as soon as after 24 h [23], comparatively to palbociclib that induces β-catenin degradation (Figures 6b and 6d). Thus, the reduction in viability caused by palbociclib on NCI-H295R cells is not a consequence of a long steroid hormone deprivation but probably results from loss of β-catenin-dependent transcription, through pRB-independent processes. Palbociclib concentrations that induced a significant viability reduction of NCI-H295R cells do not fall within a clinically attainable range in the plasma [45]. However, previous assays performed on xenograft mouse tumor tissue showed that higher palbociclib levels could be locally achieved in tumor samples (6h post-dose at 100 mg/kg could be up to 25,163 ng/g), comparatively to plasma levels [46].

**Figure 7:**
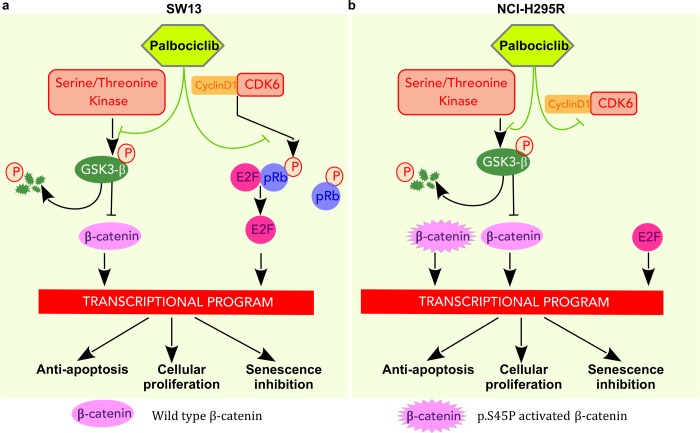
Pathways targeted by palbociclib in SW-13 and NCI-H295R cells (**a**) In SW-13 cells palbociclib acts by inhibiting the kinase activity of CDK6 and leads to a decrease in phosphorylated Rb. E2F is then sequestered and cannot activate the transcription program necessary for the G1/S transition. In addition, palbociclib leads to a decrease of phosphorylated Ser9-GSK3β, resulting in GSK3β stabilization and consecutively to the degradation of β-catenin. Consequently, these events disturb the transcription program involved in inhibition of senescence, in cellular proliferation and in avoidance of apoptosis. (**b**) In NCIH295R cells palbociclib acts by inhibiting the kinase activity of CDK6. Since the cell line is Rb^−/−^, E2F activates the transcription program necessary for G1/S transition. Palbociclib lowers Ser9-GSK3β phosphorylation, increases its stability and ultimately induces β-catenin degradation. However, this cell line carries a heterozygous mutation in the *CTNNB1* gene (p.S45P) that leads to a constitutively active form of β-catenin. Thus, only the non-mutated form of β-catenin is targeted by GSK3β that is stabilized by palbociclib.

Possible models of palbociclib action on the two cell lines used here are shown in Figure 7. In both cases, palbociclib inhibits phosphorylation of Serine9-GSK3β, that in turn induces a decrease of β-catenin signaling and changes the transcription level of its targets. These results indicate that palbociclib and ribocilib constitute potential treatments for ACCs, prompting us to test the impact of combination therapy with mitotane, a drug currently used to treat ACCs. However, only additive effects were observed when tested on the SW-13 and NCI-H295R cell lines. Since SW-13 cells have a normal amount of pRB, palbociclib also directly inhibits pRB phosphorylation, resulting in E2F inactivation and in the consequent arrest of the G1/S transition.

In conclusion, we showed that patients with high CDK6 expression levels present a poor prognosis, and are found in a unique gene expression-based cluster. They share common clinical and molecular features, such as secretion of hormones and the tendency to accumulate mutations in the Wnt/β-catenin pathway. Through its common action on the CDK6 activity and Wnt/β-catenin dependent transcription, palbociclib might be a treatment of choice for patients showing these molecular features. However, clinical assays will be necessary to verify whether ACC patients benefit from this treatment.

## MATERIALS AND METHODS

### Transcriptome and miRNome of adrenocortical carcinomas

Gene expression and miRNA expression in 79 adenocortical carcinomas were initially measured experimentally with Illumina HiSeq 2000 instruments, and treated as previously described [10]. For the present study, TCGA level 3 interpreted gene expression and miRNA expression data were downloaded from the TCGA data portal (url: https://tcga-data.nci.nih.gov/docs/publications/tcga/). Level 3 indicates that gene-level expression estimates are given as RSEM (RNA-Seq by Expectation Maximization) normalized counts. Level 3 miRNA expression-interpreted data are the miRNA transcription estimates in log2, as reads per million miRNAs mapped. Expression data from 44 adrenocortical carcinomas of French patients were downloaded from the Gene Expression Omnibus (GEO accession: GSE49278) Database. Gene expression levels were measured using the Affymetrix Human Gene 2.0 ST Array. The downloaded values of gene expression were estimated and normalized as previously described [18]. The 137 gene expression data were selected in transcriptome data files using gene symbols with Unix shell homemade scripts.

### Statistical analyses

Statistical analyses and figures were obtained using the R 3.2.2 environment [47]. Hierarchical clustering analyses and drawings of heatmaps were performed with homemade scripts using the gplots [48], vegan [49], RColorBrewer and heatmap3 [50] packages, or the ggplot2 [51] R package. Annotations of graphs were drawn using the pBrackets package. Survival analyses were performed with the stats, OIsurv and maxstat R packages. The cutoff value of expression that segregated the patients into two groups was the one that optimized the pValue of the Log-rank test. This optimization was performed using the maxstat R package.

### Reagents

Palbociclib (PD-0332991, IBRANCE®), Ribociclib (LEE-011, Kiskali®), Mitotane and PNU-74654 were purchased from CliniSciences (A8316, A8641, sc-205754 and sc-258020, respectively). Palbociclib and Ribociclib 1mM stock solutions were prepared in 100% ethanol or DMSO respectively. PNU-74654 stock solution (31.2 mM) was prepared in DMSO. Anti-CDK6 (D4S8S), CDK4 (D9G3E), Phospho-Rb (9308), GSK3-β (D5C5Z), Phospho-GSK3-β (D85E12), non-phospho-β-Catenin (D13A1) were purchased from Cell Signaling Technology. The anti-β-Catenin (MA1-301) and GAPDH (GA1R) were purchased from Thermo Fischer Scientific. The anti-ɑ-Tubulin (T9026) was purchased from Sigma.

### Cell cultures

The SW-13 (ATCC^®^ CCL-105^TM^) and NCI-H295R (ATCC^®^ CRL-2128^TM^) cell lines from ATCC were from LGC-Standards. SW-13 cells were cultured in DMEM with 4.5 g/L D-glucose, L-glutamine and pyruvate (Thermo Fisher Scientific, Life Technologies, 41966-029), supplemented with 12.5% Nu-Serum^TM^ (Corning, 355500), 1:100 ITS Premix (Corning, 354350), 100 U/mL penicillin and 100 μg/mL streptomycin (Life Technologies, 15140122). SW-13 cells were sub-cultured every three days at a 1:8 ratio. NCI-H295R cells were cultured in Dulbecco's Modified Eagle Medium (DMEM) / Nutrient mixture F-12 Ham (1:1), supplemented with GlutaMAX^TM^-I (Life Technologies, 31331-028), 2.5% Nu-Serum^TM^ (Corning), 1:100 Insulin-Transferrin-Selenium Premix (Corning, 354350), 100 U/mL penicillin and 100 μg/mL streptomycin (Life Technologies, 15140122). The subculture of NCI-H295R was carried out every four days at a 1:4 ratio. SW-13 and NCI-H295R cells were cultured in a humidified incubator with 5% CO_2_. They were seeded at a density of 5500 cells/cm^2^ and 50,000 cells/cm^2^ respectively, in 6-well or 96-well plates (TPP) for viability assays, senescence assays and protein extractions, and Petri dishes before quantitative RT-PCR experiments. Twenty-four hours after plating, palbociclib, ribociclib, 100% ethanol or DMSO was added to the cell culture, and 96 h after the addition of palbociclib or ribociclib, senescence assays, protein extracts and quantitative RT-PCR were performed as described below.

### Cellular proliferation assay

Cells were plated in 96-well plates (TPP), and incubated for 24 h, before treatment with palbociclib, ribociclib or the corresponding vehicle. After 96 h of treatment, viability was assayed using the CellTiter-Glo® Luminescent Cell Viability kit (Promega), following the manufacturer's instructions. Luminescence was measured with a SpectraMax i3 Multi-Mode Microplate Detection Platform (Molecular Devices, Sunnyvale, CA, USA). Assays were performed in duplicates, in three independent experiments.

### Cell cycle analyses by flow cytometry

Cells were plated in 6-well plates (TPP), and incubated for 24 h, before treatment with palbociclib, ribociclib or the corresponding vehicle. After 94 h of treatment, 10 μM EdU was added to the cell culture media for 2 h. The cells were then collected and washed twice with PBS. Click-it reactions were performed using the Click-iT Plus EdU Alexa Fluor 647 Flow Cytometry Kit (ThermoFischer Scientific) according to the manufacturer's recommendations. The cells were then counterstained with propidium iodide for 30 min. The cell cycle profile was generated using a CyAn ADP 9C analyser (Beckman Coulter). The analysis was performed with the Flowjo software (LLC). Cell cycle was studied on technical duplicates, in three independent experiments.

### Cortisol assay

200,000 NCI-H295R cells were plated in 24-well plates (TPP) in a volume of 500 μL of culture medium. After 24 hours, cells were treated with palbociclib, ribociclib, PNU-74654 or the corresponding vehicle. Drugs or vehicle were added in 500 μL of culture medium, to a total volume of 1 mL. After 48h of treatment, cortisol concentration was measured in the supernatant of NCI-H295R cells, using the Cortisol ELISA kit of Cayman chemical (ref: 500360), as described by the manufacturer. Before the assay, all the supernatants (except that with PNU-74654) were diluted 1:10 in fresh culture medium. Assays were performed in technical duplicates on cell media from three independent experiments.

### Measurement of apoptosis

To measure apoptosis, the cells were seeded at the previously mentioned concentration used in 96-well plates. They were drugged 24 h later and images were obtained using the Essen IncuCyte® ZOOM Live-Cell Analysis system. For the apoptosis experiments, caspase and cytotoxicity reagents (Essen Bioscience, Ltd, Welwyn, Garden City, Hertfordshire, UK) were added to the medium, resulting in a 1:1000 dilution of each reagent. Cell apoptosis and cytotoxicity of the drug were monitored for 96 h after treatment with 4 acquisitions per well every hour. Each condition was performed in triplicate. Analysis was performed using the software for the IncuCyte® ZOOM Live-Cell Analysis system. Assays were performed in duplicates, in three independent experiments.

### Senescence-associated β-galactosidase assay

After 96 h of cell treatment in 6-well plates (ATCC) with palbociclib, ribociclb or the vehicle, the senescence assay was performed using the Senescence β-Galactosidase Staining Kit (Cell Signaling, #9860), as described by the manufacturer. The cells were rinsed with Dulbecco's phosphate-Buffered Saline (Invitrogen), and fixed as described by the manufacturer. They were then incubated in 500 μL per well of X-Gal staining solution mix overnight at 37°C. Pictures of four fields (200X total magnification) of each well were taken with a Nikon Digital Sight DS-Fi1 mounted on a Nikon Eclipse TS100 microscope (Nikon France, Champigny-sur-Marne, France). Senescent cells were then counted using the ImageJ program. Assays were performed in experimental duplicates (2 wells), in three independent experiments.

### Clonogenic assay

SW-13 (5,500 cells/cm^2^) or 50,000 NCI-H295R cells/cm^2^ were plated in 6-well plates (TPP). Drugs were added to the cell-culture media 24 h later at appropriate concentrations. The cells were incubated with the drugs for 96 h, before new counting and plating at 400 cells/cm^2^ (SW-13 cells) or 5,000 cells/cm^2^ (NCI-H295R) in 6-well plates. Seven days (SW-13 cells) or 10 days (NCI-H295R) later, the cells were fixed with 4% para-formaldehyde for 5 min at room temperature and colored with 0.05% crystal violet (Sigma, C3886) diluted in water for 30 min. The clones from three independent experiments were counted.

### Western blot analysis

Cells were grown on 35 mm plates (ATCC) and protein extracted with lysis buffer containing 25 mM Tris-HCl pH 7.5, 100 mM NaCl, 1 mM EDTA, 1 mM EGTA, 0.5% NP40 (#492016) (Merck Millipore), 1% Triton^™^ X-100 (9002-93-1, Sigma Aldrich), cOmplete^™^ EDTA-free Protease Inhibitor Cocktail (Sigma Aldrich), and Phosphatase Inhibitor Cocktail Set II (524625, Merck Millipore). Proteins were separated on NuPAGE^tm^ 4-12% Bis-Tris gels (Thermo Fischer Scientific) and transferred onto nitrocellulose membranes (GE Healthcare). Primary antibodies were diluted at a final concentration of 1:1000 in Tris-buffered saline solution with 0.05% TWEEN^®^ 20. Secondary antibodies were used as recommended in the manufacturer's instructions. Relative quantifications were performed on three different western blot experiments.

### Gene expression

RNAs were extracted with the Nucleospin^®^ RNA extraction kit (Macherey-Nagel), following the manufacturer's instructions. RNA extracts (2 μg) were treated with 0.1 U/μL of DNAse I (Thermo Fischer Scientific), in a total volume of 20 μL at 37°C for 30 min. DNase I was then inactivated by heating at 65°C for 10 min after addition of 2 μL of 50 mM EDTA (Thermo Fischer Scientific). Reverse transcription of 1 μg of DNase I-treated RNA was performed using 15 ng/μL random primers (Invitrogen), 1 U/μL Ribolock RNase inhibitor, 1 mM of each dNTP and 10 U/μL RevertAid reverse transcriptase (Thermo Fisher Scientific), in a final volume of 20 μL. The reaction was performed in an Eppendorf Mastercycler thermocycler machine (Eppendorf France, Montesson, France), for 5 min at 25°C and then 60 min at 42°C. Reaction was stopped by heating at 70°C for 5 min. Quantitative PCRs were performed with 1 μL of 1/5 diluted first strands of cDNA in a total volume of 15 μL of 1X ABsolute SYBR Capillary Mix (Thermo Fisher Scientific, AB-1285), with 73 nM of each PCR primer. Reactions were performed in 20 μL LightCycler capillaries (Roche). Primer sequences were as follows: AXIN2-F 5′-GCTGACGGATGATTCCATGT-3′, AXIN2-R 5′-ACTGCCCACACGATAAGGAG-3′[52], ACTB-F 5′-GAGCTACGAGCTGCCTGAC-3′ and ACTB-R 5′-GCACTGTGTTGGCGTACAG-3′. PCR amplification was performed with a LightCycler 1.5 thermocycler and analyzed with LightCycler Software 3.5 (Roche, Boulogne-Billancourt, France). Cycling conditions were as follows: initial denaturation of enzyme 95°C for 15 minutes, and 50 amplification cycles (95°C for 15 sec, 60°C for 30 sec and 72°C for 20 sec) before annealing of all samples and gradual temperature increase to 95°C to trace the melting curve. Assays were performed in technical triplicates on RNAs extracted from three independent experiments.

## SUPPLEMENTARY MATERIAL



## Abbreviations

ACC: Adrenocortical carcinoma
COSMIC: Catalogue of Somatic Mutations In Cancers
EdU: 5-ethynyl-2′-deoxyuridine
FDA: Food and Drug Administration
GEO: Gene Expression Omnibus
HR: Homologous Recombination
IC50: half maximal inhibitory concentration
OS: Overall Survival
RFS: Relapse Free Survival
TCGA: The Cancer Genome Atlas
KEGG: Kyoto Encyclopedia of Genes and Genomes
RSEM: RNA-Seq by Expectation-Maximization.
