# Supplementary material for: A hypothesis-driven approach identifies CDK4 and CDK6 inhibitors as candidate drugs for treatments of adrenocortical carcinomas
Source: Aging (Albany NY). 2017 Dec 26;9(12):2695–716. doi: 10.18632/aging.101356 (PMC5764399; doi:10.18632/aging.101356)
Supplement: Supplementary file 1 [file aging-09-2695-s001.pdf]

SUPPLEMENTARY MATERIAL

Please browse the link in Full text version to see

Supplementary Table 1. Association of 136 genes involved in G1/S phase transition, DNA replication and DNA damage response with overall survival and relapse free survival) in ACC patients of the TCGA consortium.

Data from n=54 and n=79 ACC samples were used for the log-rank correlation test for RFS and OS, respectively. Log-Rank tests the difference of RFS or OS time between "High" and "Low" expression groups of patients. The cutoff is the value of gene expression maximizing the significance of the difference between those two groups. The percentile is the proportion of individuals below the cutoff value. Adjusted *p* (Adj *p*) values have been obtained following the Benjamini Hochberg method. Significant Adj *p* values are written in bold characters. The correlation with *MKI67* gene expression value was tested with Pearson test. Correl. coef. is the Pearson product-moment correlation coefficient that estimates the correlation of the expression level of each gene and of *MKI67*.

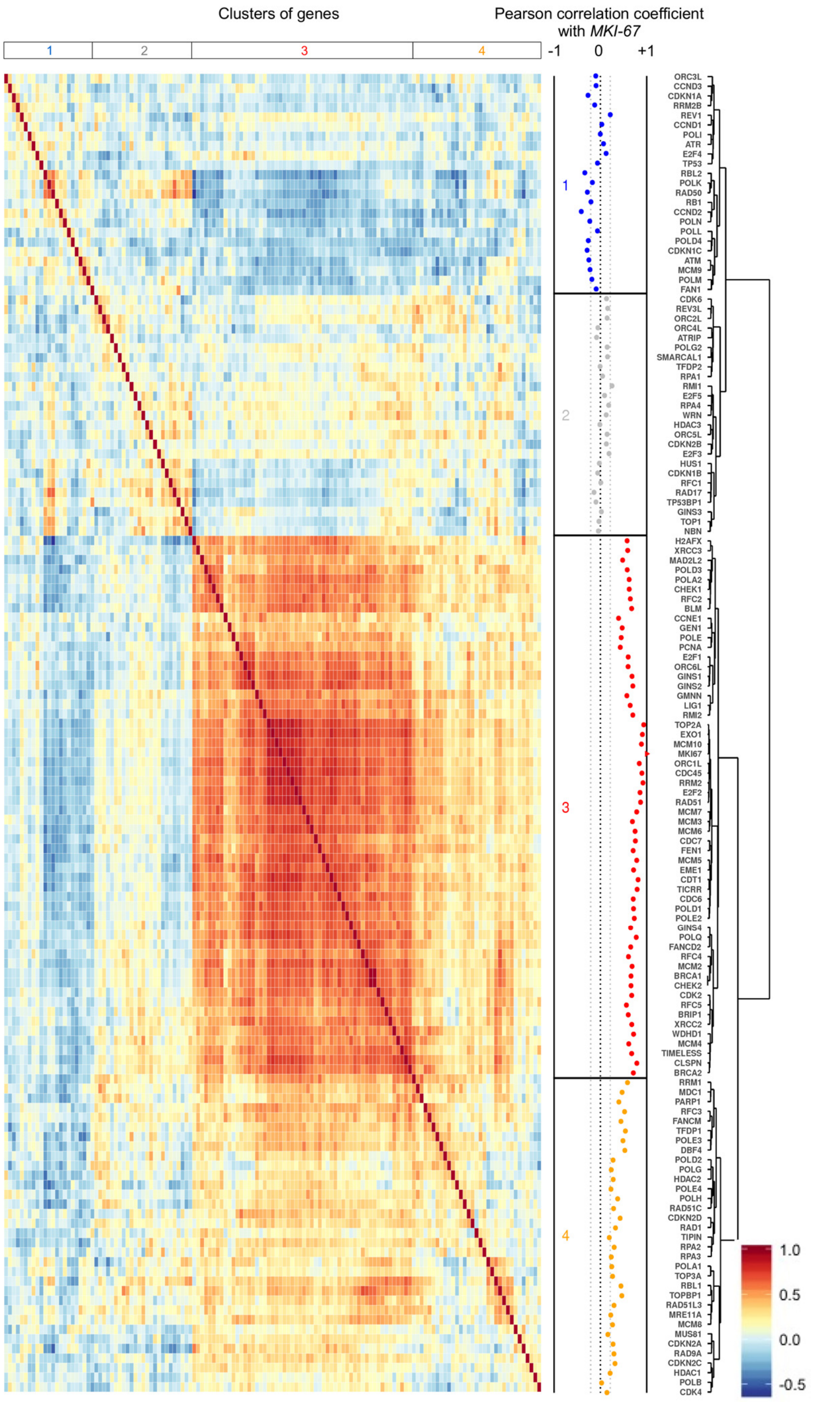

Supplementary Figure 1 Pearson correlation coefficient-based heatmap representing the similarity of 137 gene expression values in 79 ACCs. The 137 genes (right side) are involved in G1/S transition, and in DNA replication and repair. Colors indicate the Pearson correlation coefficient values between genes, as indicated by the color scale at the bottom-right. Dissimilarities between clusters are indicated by the dendrogram (right side). Hierarchical clustering of genes based on Pearson correlation coefficient values resulted in four clusters of genes, as indicated in the dot plot at the right side, and at the top of the heatmap. In the dot plot, dots indicate the Pearson correlation coefficient values between each gene and *MKI67*. Grey colored dashed lines indicate the threshold correlation coefficient values for a significant Pearson correlation test (+/- 0.21).

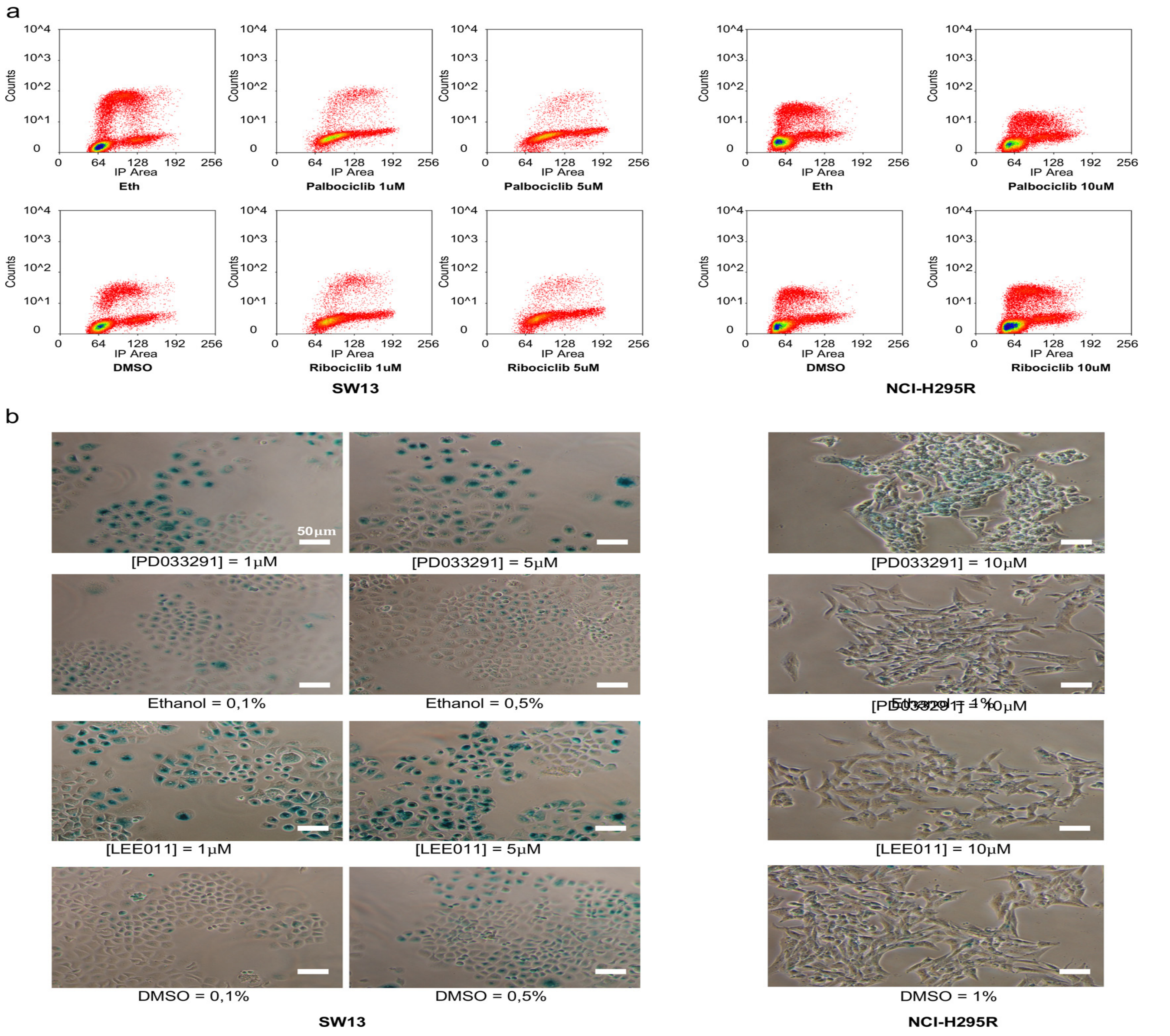

Supplementary Figure 2. Palbociclib and ribociclib impacts on cell cycle and senescence in NCI-H295R and SW-13 cells. (**a**) Bivariate plots showing DNA content (propidium iodide staining, x axis) and EdU incorporation (Alexa Fluor® 647 Staining, y axis) for SW-13 cells (left panels) and NCI-H295R cells (right panels) upon treatment with palbociclib, ribociclib or mock-treated cells. (**b**) Images showing β-galactosidase activity staining (senescence marker) in SW-13 cells (left panels) and in NCIH-295R cells (right panels) upon treatments with palbociclib, ribociclib or in mock-treated cells. Magnification scale bars indicate 50 µm.
